# Exploring the mechanism of sesamin for the treatment of PM_2.5_-induced cardiomyocyte damage based on transcriptomics, network pharmacology and experimental verification

**DOI:** 10.3389/fphar.2024.1486563

**Published:** 2024-11-05

**Authors:** Yadong Zhang, Rui Wen, Jingyi Ren, Fan Zhang, Huanting Pei, Jinshi Zuo, Yuxia Ma

**Affiliations:** Department of Nutrition and Food Hygiene, School of Public Health, Hebei Medical University, Hebei Key Laboratory of Environment and Human Health, Shijiazhuang, China

**Keywords:** sesamin, PM_2.5_, heart injury, ferroptosis, network pharmacology, transcriptomics, ACSL4

## Abstract

**Introduction:**

Exposure to fine particulate matter (PM_2.5_) is known to be associated with cardiovascular diseases. Sesamin (Ses) is a natural phenolic compound found in sesame seeds and sesame oil. Ferroptosis is a novel mode of cell death characterised by iron-dependent lipid peroxidation. This study aims to explore whether PM_2.5_ can induce ferroptosis in H9C2 cells and to investigate the precise protective mechanism of Ses.

**Methods:**

Based on transcriptomic data, PM_2.5_ may induce ferroptosis in cardiomyocytes. The ferroptosis inducer erastin and ferroptosis inhibitor ferrostatin-1 (Fer-1) were used to illustrate the mechanisms involved in PM_2.5_-induced H9C2 cell injury. Using network pharmacology, the pharmacological mechanism and potential therapy targets of Ses were explored for the treatment of PM_2.5_-induced cardiomyocyte injury. H9C2 cells were cultured and pretreated with Fer-1 or different concentrations of Ses, and then cardiomyocyte injury model was established using erastin or PM_2.5_. Indicators of oxidative responses, including total superoxide dismutase, reduced glutathione, glutathione peroxidase and malondialdehyde, were measured. The expression levels of ferroptosis-related proteins were determined through Western blot analysis.

**Results:**

Results demonstrate that PM_2.5_ induces ferroptosis in H9C2 cells and Ses exerts a protective effect by suppressing ACSL4-mediated ferroptosis.

**Discussion:**

Overall, these findings elucidate a novel mechanism by which Ses ameliorates the detrimental effects of PM_2.5_ on cardiomyocytes.

## Introduction

Environmental pollution has gradually become an urgent problem that must be addressed with the development of science and the economy. Data from the World Health Organisation (WHO) revealed that a substantial proportion, reaching up to 92%, of the global population resides in regions where air pollution levels exceed established thresholds. Fine particulate matter (PM_2.5_), a prominent constituent of atmospheric contaminants, has been associated with major adverse health effects. PM_2.5_ refers to microscopic particulates with an aerodynamic diameter of less than 2.5 μm (Bright et al., 2023). The WHO indicates that approximately 4.2 million deaths are attributed to PM_2.5_ exposure each year (Morishita et al., 2018). In China, approximately 1.2 million premature deaths were caused by high levels of PM_2.5_ in 2010 (Xie et al., 2016). The persistent presence and widespread distribution of PM_2.5_ have substantial implications for human health and the atmospheric environment (Gao et al., 2022). Growing evidence indicates that PM_2.5_ leads to adverse effects on the cardiovascular and respiratory systems (Kim et al., 2022; Li et al., 2022). Huang et al. (2019) provide evidence that chronic exposure to relatively high concentrations of PM_2.5_ is positively correlated with stroke and its major subtypes. Liang et al. (2021) found that exposure to specific concentrations of PM_2.5_ in a monsoon climate region notably raised the risk of emergency room visits for patients with atrial fibrillation (AF). Additionally, abundant evidence shows a strong association between PM_2.5_ and atherosclerosis (Woo et al., 2021; Kwok et al., 2023; Macchi et al., 2023). Therefore, understanding the mechanism by which PM_2.5_ causes cardiomyocyte damage and developing effective prevention and treatment strategies are imperative.

Since the 1990s, the potential impact of particulate matter in eliciting health effects through oxidative reactions has been recognised. Extensive efforts have been made to clarify the mechanisms underlying PM_2.5_-induced adverse health effects. With the deepening of research, many new mechanisms of PM-induced toxicity share a common trigger: PM-induced oxidative stress (Vilas-Boas et al., 2024). Oxidative stress is an underlying cause of severe illnesses due to the oxidant–antioxidant imbalance. Reactive oxygen species (ROS) and cellular oxidative stress are considered inducers of diseases (Villarreal et al., 2020; Xiao et al., 2020). Ferroptosis, a novel type of programmed cell death characterised by lipid peroxidation, relies on iron-mediated oxidative damage (Dong et al., 2023). Several studies have shown that ferroptosis is involved in CVDs. Suppression of ferroptosis has a beneficial effect on cardiovascular injuries by alleviating mitochondrial damage and endothelial injury (Wang et al., 2020; Cai et al., 2022; Luo et al., 2022). Based on these studies, inhibiting oxidative stress and ferroptosis may be an effective therapeutic strategy to reduce the risk of adverse health effects caused by air pollution.

Growing evidence indicates that the relationship between human health and air pollution is influenced by dietary intake. Previous studies have shown that some active constituents in plant extracts, such as procyanidin and resveratrol, can substantially alleviate PM_2.5_-induced cardiovascular damage (Chen et al., 2022; Yin et al., 2023). However, these active constituents are difficult to obtain in sufficient doses directly from food and are often consumed in the form of dietary supplements. Ses, a natural lignin-like compound separated from sesame seeds and sesame oil, has attracted widespread attention due to its extensive biological functions, including antioxidant and anti-inflammatory activities (Dalibalta et al., 2020; Majdalawieh et al., 2022). A meta-analysis indicated that daily dietary supplementation of 20–200 mg of Ses (approximately 4–40 g sesame seeds) can produce major cardiovascular benefits (Sun et al., 2022). Given the relative ease of obtaining Ses from dietary sources compared to other active constituents, its potential health benefits for the cardiovascular system warrant more extensive and rigorous investigation. At present, research evidence on the protective effect of Ses on PM_2.5_-induced cardiomyocyte injury is still lacking. This study aimed to elucidate the mechanism of PM_2.5_-induced cardiomyocyte damage and further explore the protective effects and targets of Ses in a PM_2.5_-induced cardiomyocyte injury model.

## Materials and methods

### Bioinformatic analysis

The GSE dataset (GSE211949) was downloaded from NCBI GEO DataSets (https://www.ncbi.nlm.nih.gov/geo/). This dataset, generated using the GPL24247 platform, includes gene expression profiles from three normal mouse cardiomyocyte cells and 3 mouse cardiomyocyte cells treated with PM_2.5_ at a concentration of 100 μg/mL. Differentially expressed genes (DEGs) were identified using the R package DESeq2 (version 1.40.2). A |log_2_FoldChange|≥1.5 and adjusted *p <* 0.05 were set as the screening criteria. Pearson correlation coefficients were calculated using the cor. test function in R (version 4.0.3). Principal component analysis (PCA) was conducted using the FactoMineR package. The volcano plot and heatmap were generated using the “ggplot2” and “pheatmap” packages in R, respectively. The “clusterProfiler” package in R was used to perform Kyoto Encyclopaedia of Genes and Genomes (KEGG) enrichment analysis on the DEGs. The significance of enrichment analysis results was determined by a *p*-value below 0.05.

### Computational pharmacology prediction

The candidate targets of Ses were predicted using Swiss Target Prediction (http://swisstargetprediction.ch/), Bioinformatics Analysis Tool for Molecular mechanism of Traditional Chinese Medicine (BATMAN-TCM, http://bionet.ncpsb.org.cn/batman-tcm/) (Liu et al., 2016), TargetNet (http://targetnet.scbdd.com/calcnet/index/) and Traditional Chinese Medicine Systems Pharmacology (TCMSP, https://www.tcmsp-e.com/#/home). Targets from the four aforementioned databases were synthesised to remove duplicate values and obtain the Ses-related targets. After organising the data, the VennDiagram package (version 1.7.3) in R software was used to find the intersection between the DEGs and the Ses targets. Subsequently, KEGG enrichment analysis was performed for the genes at the intersection. PPI analysis was conducted using the STRING database (STRING v11.5, https://cn.string-db.org) (Szklarczyk et al., 2019), and PPI networks were analysed using Cytoscape (version 3.9.1). The cytoHubba plugin was used to calculate clustering coefficient scores, which were ranked from high to low.

### Molecular docking analysis

The 2D molecular structure of the ligand, Ses, was obtained from the PubChem database (https://pubchem.ncbi.nlm.nih.gov/), and the AlphaFold structure of ACSL4 (receptor) was retrieved from the UniProt database (https://www.uniprot.org/). The 2D structure of Ses was converted into a 3D configuration using ChemBio3D Ultra (version 21.0.0.28), and the MM2 force field was used to optimise energy. Hydrogen atoms and charges were added to the ligand and receptor files using AutoDock Tools (version 1.5.7), and the files were subsequently exported in PDBQT format. After processing the structures of Ses and ACSL4, molecular docking was performed using AutoDockVina (version 1.1.2) (Trott and Olson, 2010) and visualised with PyMOL software (version 2.5).

### Materials and reagents

H9C2 cell lines were supplied by Solarbio Science & Technology Co., Ltd. (Beijing, China). The PM_2.5_ was collected using a membrane collection method on the Hebei Medical University campus. Ses (HPLC>98%) was purchased from Shanghai Aladdin Biochemical Technology Co., Ltd., (Shanghai, China). DMEM high-glucose medium, foetal bovine serum (FBS) and pancreatic digestive fluid (containing EDTA) were obtained from ZETA LIFE Inc. (CA, United States). Dimethyl sulfoxide (DMSO) and the ROS kit were purchased from Shanghai Biyotime Bio-technology (Shanghai, China). RIPA lysate, phosphate buffer (PBS), Phenylmethanesulfonyl fluoride protease inhibitor, skim milk powder, ECL Western blotting Substrate (ELC Plus), Tris-Base, Tween-20 and Fe^2+^ kit were purchased from Solarbio Science (Beijing, China). Anti-ACSL4 antibody, anti-β-actin polyclonal antibody, anti-GPX4 antibody and anti-SLC7A11 antibody were purchased from ABclonal Biotech Co., Ltd. (Wuhan, China). MDA, SOD, LDH, GSH, GSH-Px and BCA kits were purchased from Nanjing Jiancheng Bioengineering Institute (Nanjing, China). The C11 BODIPY^581/591^ and JC-1 fluorescence probes were purchased from MedChemExpress (Shanghai, China). The CCK-8 kit was obtained from ZETA LIFE Inc. (CA, United States).

### Cell culture

H9C2 cells were cultured in DMEM high-glucose medium containing 10% FBS and 1% dual antibiotics (containing 100 U/mL penicillin and 100 mg/mL streptomycin) at 37°C in a 5% CO_2_ incubator.

### PM_2.5_ collection and preparation

The collection and preparation of PM_2.5_ sample were performed in accordance with our previous study (Zhang et al., 2021). Briefly, PM_2.5_ samples were collected using an air sampler, and the collected PM_2.5_ quartz fibre filters were sectioned into small fragments, suspended in PBS and sonicated for 30 min. After filtration through gauze, the PM_2.5_ suspension was subjected to vacuum freeze-drying for 48 h to obtain PM_2.5_ dry powder, which was then stored at −80°C. The detailed chemical components of PM_2.5_ have also been reported in our previous study.

### Cell viability assay

CCK-8 kits were used to assess cell viability in this study. The H9C2 cell line was plated in 96-well plates at a density of 5 × 10^3^ cells per well and cultured in 10% FBS medium for 12 h. Cells were then treated with different concentrations of Ses or PM_2.5_ for 24 h. The CCK-8 solution was added to the culture medium at a ratio of 1:10 in each well. Cells were incubated at 37°C in a 5% CO_2_ atmosphere for an additional 1 h. After the treatment, absorbance was measured using an enzyme-labelling instrument at 450 nm.

### Determination of MDA, GSH and Fe^2+^

Multiple oxidative stress markers (MDA, SOD and GSH) and Fe^2+^ levels in H9C2 cells were measured using assay kits according to the manufacturer’s instructions.

### Lipid peroxidation assay

The H9C2 cell line was plated in 6-well plates at a density of 3 × 10^5^ cells per well and cultured in 10% FBS medium for 12 h. Drug intervention was applied after 12 h of cultures. Lipid ROS levels and mitochondrial membrane potential were measured by flow cytometry to determine whether PM_2.5_ induced ferroptosis in H9C2 cells.

### Western blot

The expression levels of ferroptosis-related proteins, including GPX4, SLC7A11, ACSL4 and LPCAT3, were measured by Western blot. Total protein from H9C2 cells was extracted using RIPA buffer containing protease inhibitors. The supernatant from the H9C2 cells was collected and subsequently denatured by boiling in 5× loading buffer for 5 min. After denaturation, equal amounts of proteins from each sample were electrophoresed by 10%–12% SDS–PAGE and transferred to a PVDF membrane. The membrane was then blocked in 5% skim milk for 2 h. Primary antibodies were incubated overnight at 4°C. The membrane was washed three times with TTBS, each wash lasting 10 min. Subsequently, the membrane was incubated with the secondary antibody for 40 min at room temperature. Finally, protein bands were detected using enhanced chemiluminescence.

### Statistical analysis

The study analysis was performed using SPSS and GraphPad Prism. Data are expressed as the means ± SEM. Multiple group comparisons were conducted using one-way analysis of variance (one-way ANOVA). Differences were considered statistically significant when *p*-values were less than 0.05 (*p <* 0.05).

## Results

### PM_2.5_ induces ferroptosis in cardiomyocyte cells at the transcriptional level

**FIGURE 1:**
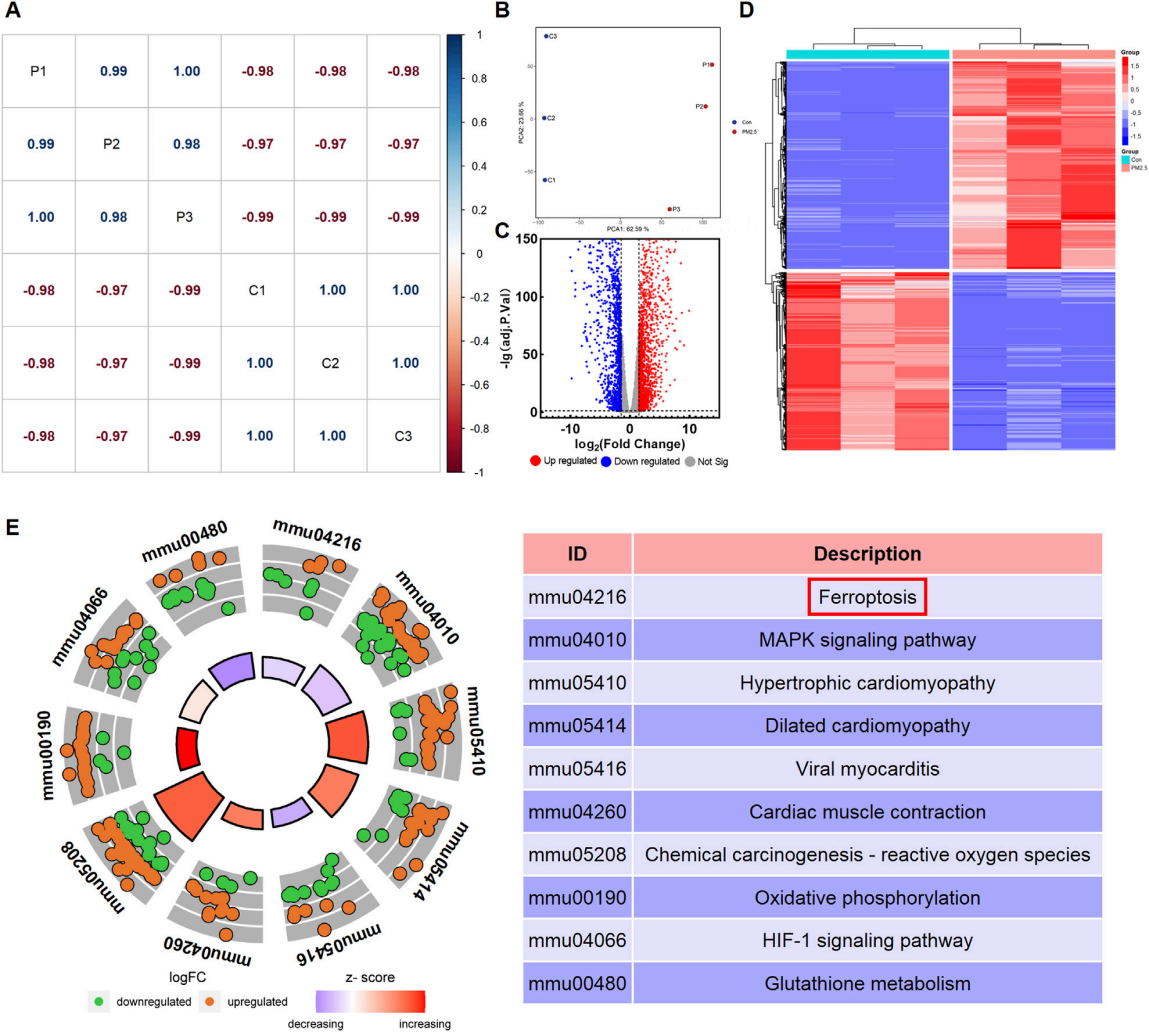
PM_2.5_ exposure altered the transcriptomics profile of HL-1 cell model. **(A)** Correlation analysis among sequencing samples. P represents PM_2.5_-exposure group samples, and C represents control group samples. **(B)** Principal component analysis (PCA) between control group (Con) and PM_2.5_-exposure group (PM_2.5_). **(C)** Volcano plots of the differentially expressed genes (DEGs). **(D)** Heatmap of the DEGs. **(E)** Kegg enrichment analysis of the DEGs.

A correlation analysis was conducted to investigate the associations amongst different samples. A correlation coefficient close to one indicates a stronger positive correlation, whilst one close to −1 signifies a stronger negative correlation. Conversely, a correlation coefficient near 0 indicates a negligible or non-existent correlation between the two samples. The results indicate a strong positive correlation amongst samples within the same group and a strong negative correlation between samples from different groups (Figure 1A). The results of the unsupervised PCA demonstrated a distinct separation between the control group and the PM_2.5_ exposure group samples. Principal component 1 (PC1) and principal component 2 (PC2) accounted for 62.59% and 23.66% of the total variance, respectively (Figure 1B). Differential expression analysis was performed using the ‘DESeq2’ R package to identify DEGs. The number of DEGs was illustrated using a volcano plot. A total of 3,212 DEGs were identified, meeting the threshold setting (|log_2_FoldChange|≥1.5 and adjusted *p <* 0.05). Amongst these DEGs, 1729 genes were upregulated, and 1,483 genes were downregulated (Figure 1C). Subsequently, a heatmap of DEGs was presented in Figure 1D. Cluster analysis revealed the expression patterns of upregulated and downregulated genes across the samples. In the heatmap, red indicates upregulation, and blue indicates downregulation. KEGG enrichment analysis was used to determine the functions of DEGs. As illustrated in Figure 1E, the DEGs were primarily involved in ferroptosis, MAPK signalling pathway, hypertrophic cardiomyopathy, dilated cardiomyopathy, viral myocarditis, cardiac muscle contraction, chemical carcinogenesis-reactive oxygen species, oxidative phosphorylation, HIF-1 signalling pathway and glutathione metabolism. The detailed information of the DEGs involved in ferroptosis were shown in Supplementary Table S1.

### PM_2.5_ induces ferroptosis in H9C2 cells

**FIGURE 2:**
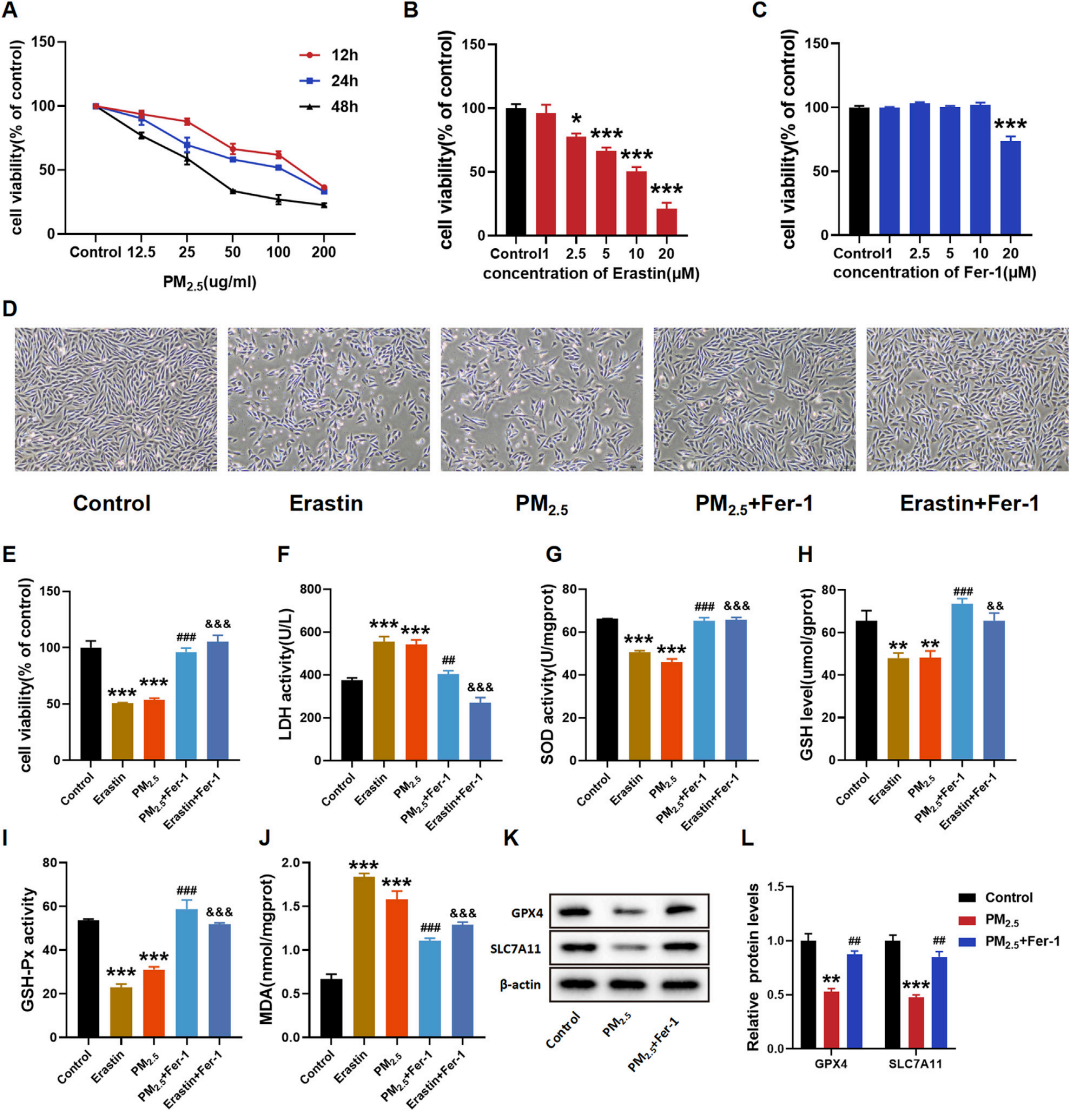
PM_2.5_ exposure induced ferroptosis in H9C2 cells. **(A–C, E)** CCK8 was used to test the cell viability of each group of cells. **(D)** Morphology and growth of H9C2 cells under optical inverted microsope. **(F–J)** LDH, SOD, GSH, GSH-Px, MDA levels were analyzed by kits. **(K–L)** GPX4 and SLC7A11 levels were examined by Western blot. The data were expressed as mean ± SEM. All datas were obtained from three replicate experiments. *p < 0.05, *versus* the Control. **p < 0.01, *versus* the Control. ***p < 0.001, *versus* the Control. ^##^ *p* < 0.01, *versus* the PM_2.5_ group. ^###^ *p* < 0.001, *versus* the PM_2.5_ group. ^&&^ *p* < 0.01, *versus* the Erastin group. ^&&&^ *p* < 0.001, *versus* the Erastin group.

H9C2 cells were treated with different concentrations of PM_2.5_ for varying durations to determine the appropriate treatment time. Figure 2A shows a notable decrease in cell viability with increasing PM_2.5_ concentration.

After exposure to PM_2.5_, a decline in cell viability was observed, with the decrease being concentration-dependent. Notably, this decline was more pronounced after 24 h of exposure compared to 12 and 48 h of exposure. The results indicated that the half inhibition concentration (IC50) of PM_2.5_ was close to 100 μg/mL (Figure 2A). The IC 50 value of erastin and the optimal Fer-1 dose in H9C2 cells were then measured to determine the dose for subsequent experiments (Figures 2B,C, respectively). The H9C2 cell viability in Figure 2B exhibited a notable concentration-dependent decrease in response to erastin. In addition, at concentrations up to 10 μΜ, Fer-1 did not demonstrate remarkable cytotoxicity (Figure 2C). Cell morphology, cell viability and LDH release assays confirmed the damaging effects of PM_2.5_ exposure, and Fer-1 can attenuate these changes (Figures 2D–F). Subsequently, the oxidative stress indices, including SOD, GSH, GSH-Px and MDA, were examined (Figures 2G–J). As expected, exposure to PM_2.5_ remarkably decreased the levels of SOD, GSH and GSH-Px whilst increasing the level of MDA. However, Fer-1 substantially reversed these changes and alleviated oxidative stress. As two important biomarker proteins of ferroptosis, GPX4 and SLC7A11 exhibited relatively decreased expressions due to PM_2.5_ exposure. In contrast, Fer-1 pretreatment remarkably increased the expressions of GPX4 and SLC7A11 (Figures 2K, L).

### Pharmacological prediction of ses for mitigating cardiomyocyte cell injury induced by PM_2.5_

**FIGURE 3:**
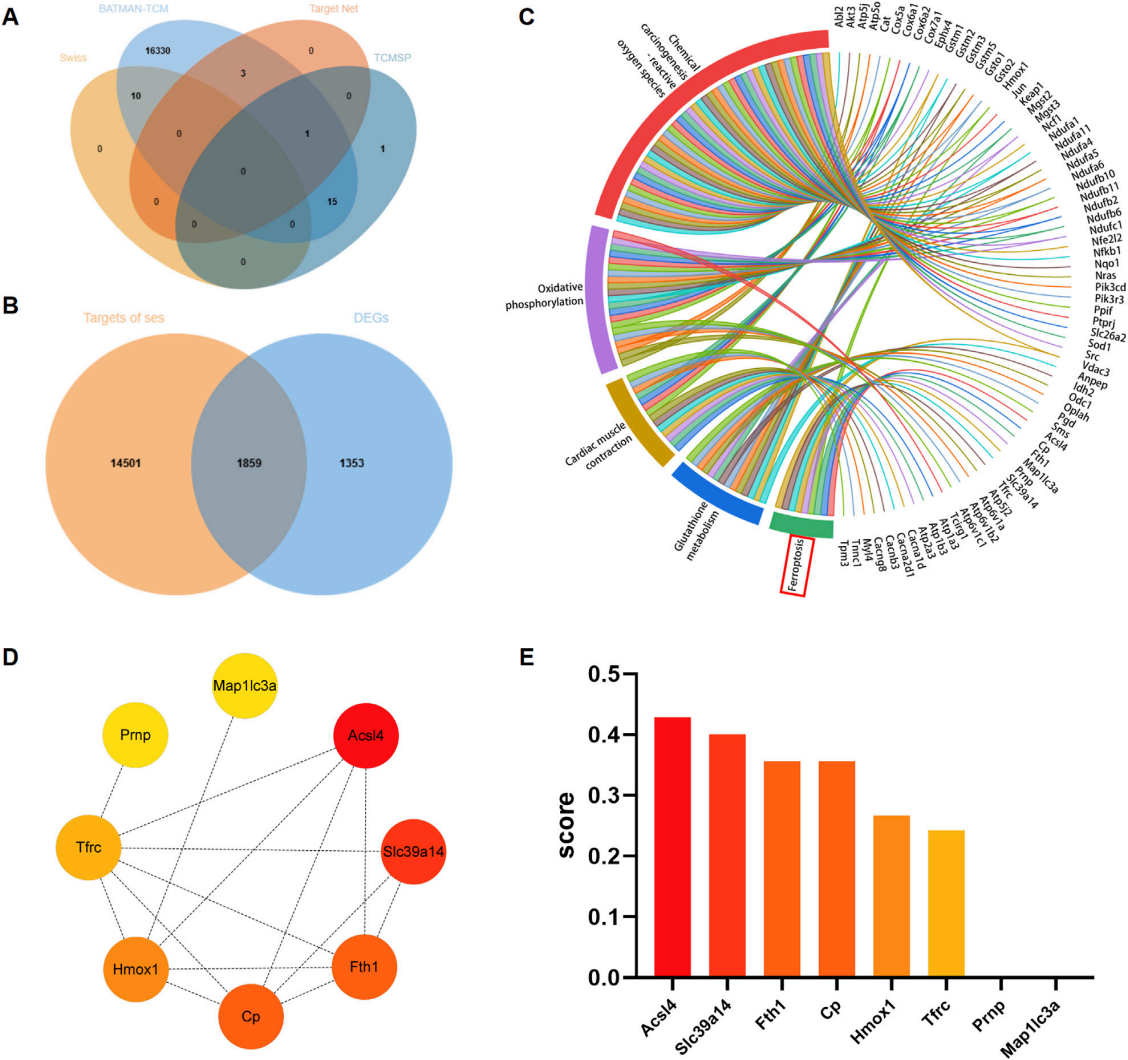
Analysis of core targets of sesamin (Ses) treatment for PM_2.5_-induced ferroptosis in H9C2 cells based on network pharmacology. **(A)** Venn graph exhibiting the numbers of predicted Ses targets. **(B)** Venn diagram showing the interaction of Ses’s targets and the differentially expressed genes (DEGs). **(C)** KEGG enrichment analysis of the interaction genes between Ses’s targets and DEGs. **(D)** The enriched genes within the ferroptosis pathway were visualized through a protein-protein interaction network to identify hub genes. **(E)** The clustering coefficient scores of genes in the network were visualized via a histogram.

A total of 16,360 targets of Ses were obtained from the Swiss Target Prediction, BATMAN-TCM, TargetNet and TCMSP databases, following the removal of partially overlapped targets. A total of 10 targets were obtained from Swiss Target Prediction, 16,359 targets from BATMAN-TCM, 4 targets from TargetNet and 17 targets from TCMSP (Figure 3A). According to Figure 3B, a total of 1859 targets were identified by integrating the Ses targets with the DEGs, namely, the targets of Ses involved in the treatment of PM_2.5_-induced cardiomyocyte cells injury. Subsequently, KEGG enrichment analysis of the aforementioned intersection targets was conducted. The results showed that ferroptosis, chemical carcinogenesis-reactive oxygen species, glutathione metabolism, oxidative phosphorylation and cardiac muscle contraction were substantially enriched (Figure 3C). The PPI network was further constructed based on the genes enriched in ferroptosis and ranked by the clustering coefficient algorithm from high to low using the cytoHubba plugin (Figures 3D, E). The results revealed that ACSL4 had the highest score, followed by Slc39a14, Fth1, Cp, Hmox1, Tfrc, Prnp and Map1lc3a.

### Ses attenuates ferroptosis caused by erastin in H9C2 cells

**FIGURE 4:**
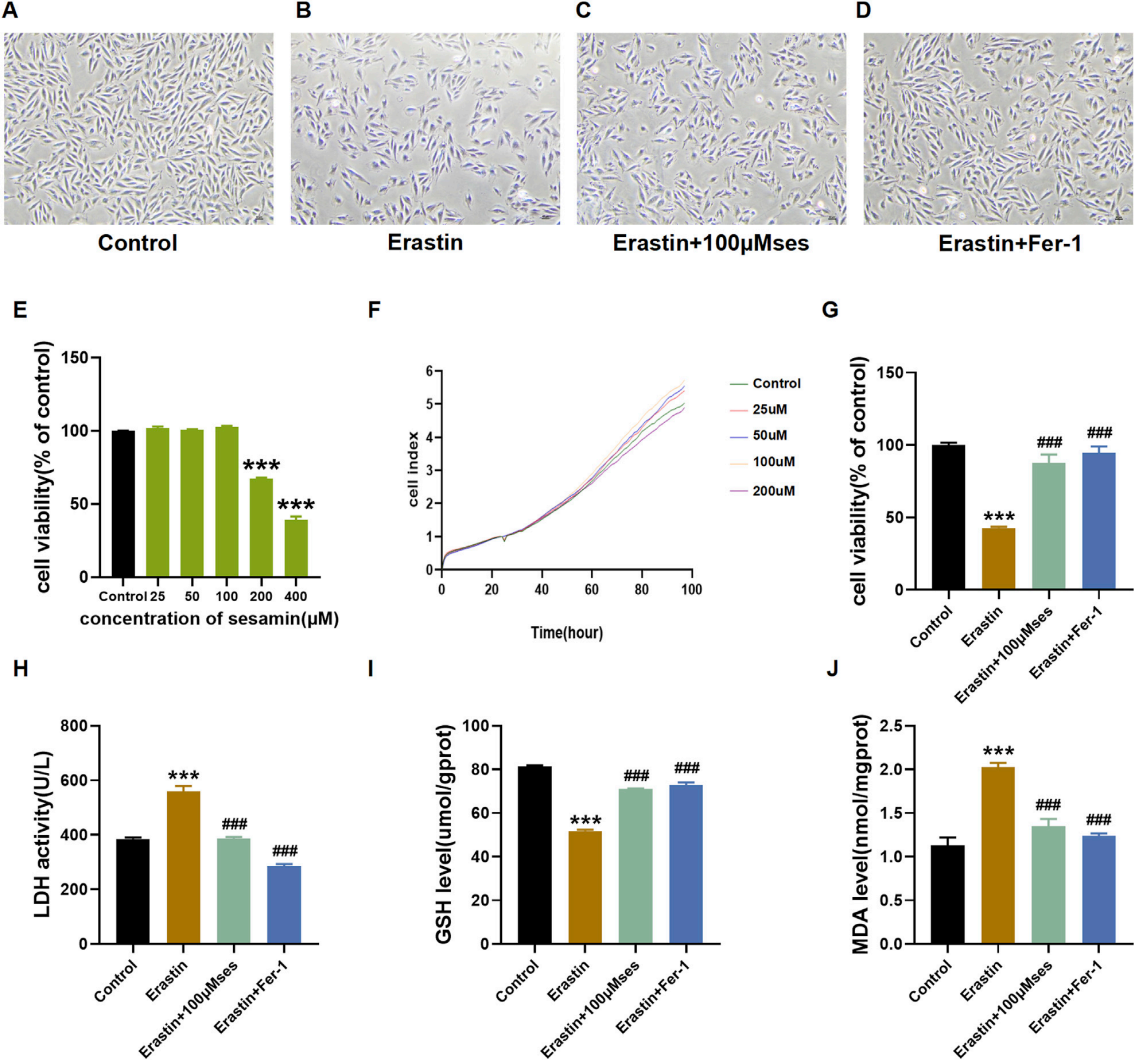
Ses attenuates ferroptosis caused by erastin **(A–D)** Morphology and growth of H9C2 cells under optical inverted microsope. **(E–G)** CCK8 and RTCA ICELLigence system were used to test the cell viability. **(H–J)** LDH, GSH, MDA levels were analyzed by kits. The data were expressed as mean ± SEM. All datas were obtained from three replicate experiments. ***p < 0.001, *versus* the Control. ^###^ *p* < 0.001, *versus* the Erastin group.

As shown in Figures 4A–D, after treatment with erastin, the cells shrank and lost their normal shape. However, pretreatment with Ses drastically inhibited this damage. H9C2 cells were treated with different concentrations of Ses (25–400 μM), and a CCK-8 assay was used to determine the viability of H9C2 cells. The results indicated no remarkable cytotoxicity of Ses at concentrations up to 100 μΜ (Figure 4E). Hence, concentrations of Ses, which include 25, 50 and 100 μΜ, were chosen as the intervention concentrations for subsequent experiments. An iCELLigence device was also used to monitor the cell index in real time (Figure 4F). The trend was consistent with the results of the CCK-8 assay. Based on the results of the CCK-8 assay and LDH release assays, Ses noticeably weakened the cellular damage caused by erastin (Figures 4G, H). Similarly, a decrease in the levels of GSH and an increase in the levels of MDA were observed after the administration of erastin. However, these alterations can be reversed by pretreatment with Ses (Figures 4I, J). The abovementioned results indicate that Ses is a potential candidate ferroptosis inhibitor and attenuates ferroptosis caused by erastin in H9C2 cells.

### Ses attenuates cell injuries caused by PM_2.5_ in H9C2 cells

**FIGURE 5:**
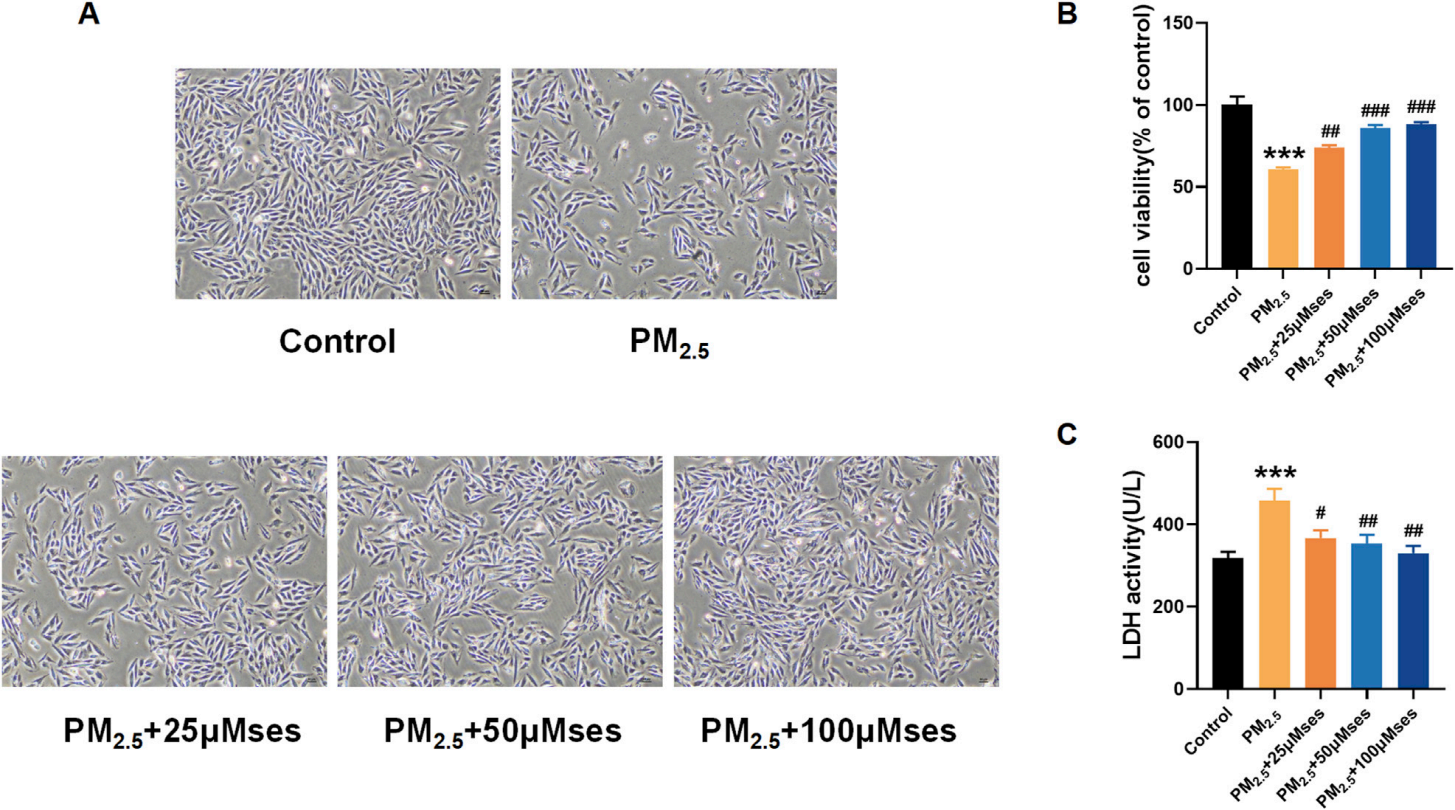
Ses moderates damages caused by PM_2.5_ in H9C2 cells **(A)** Morphology and growth of H9C2 cells under optical inverted microsope. **(B)** CCK8 was used to test the cell viability of each group of cells. **(C)** LDH level was analyzed by kits. The data were expressed as mean ± SEM. All datas were obtained from three replicate experiments. ***p < 0.001, *versus* the Control. #*p* < 0.05, *versus* the PM_2.5_ group. ^##^ *p* < 0.01, *versus* the PM_2.5_ group. ^###^ *p* < 0.001, *versus* the PM_2.5_ group.

Figures 5A–C illustrate the protective effect of different concentrations of Ses on cell morphology, cell viability and LDH release. A dose-dependent phylactic effect of Ses pretreatment was also observed.

### Ses attenuates oxidative stress and mitochondrial damage caused by PM_2.5_ in H9C2 cells

**FIGURE 6:**
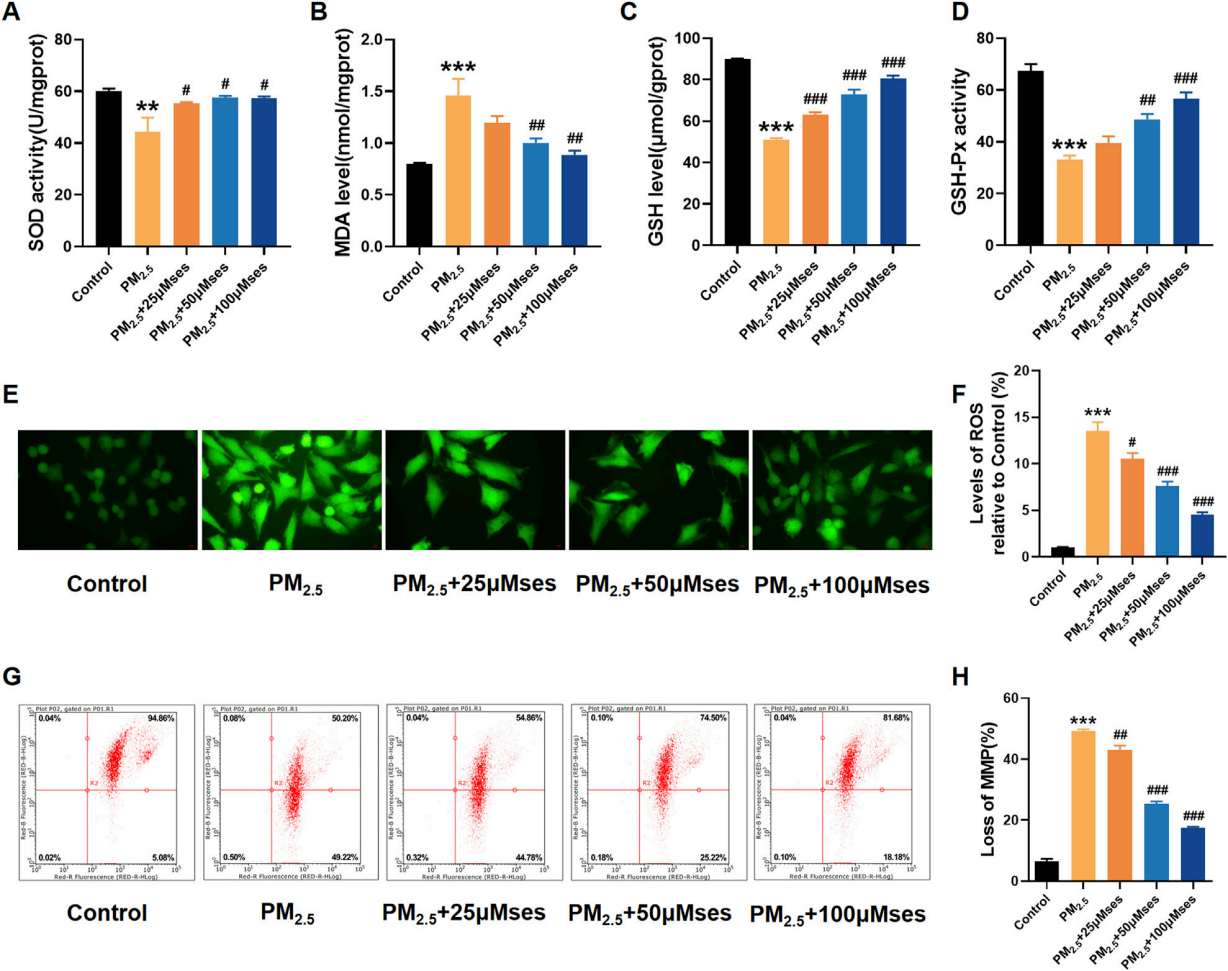
Ses weakens oxidative stress and mitochondrial damage caused by PM_2.5_ in H9C2 cells. **(A–D)** SOD, MDA, GSH, GSH-Px levels were analyzed by kits. **(E)** The contents of ROS were measured by DCFH-DA. **(F)** Quantitative analyses of ROS. **(G)** Detection of MMP by flow cytometry JC-1 staining. **(H)** Quantitative analyses of JC-1. The data were expressed as mean ± SEM. All datas were obtained from three replicate experiments. **p < 0.01, *versus* the Control. ***p < 0.001, *versus* the Control. ^#^ *p* < 0.05, *versus* the PM_2.5_ group. *p* < 0.01, *versus* the PM_2.5_ group. ^###^ *p* < 0.001, *versus* the PM_2.5_ group.

Concomitant with the progressive impairment of cardiomyocyte integrity, various markers of oxidative stress, such as SOD, GSH and GSH-Px, exhibited a notable decrease. However, with increased treatment levels of Ses, the levels of these crucial ROS scavengers were upregulated (Figures 6A, C, D). The MDA level, as the end product of lipid peroxidation, was downregulated after Ses administration (Figure 6B). The DCFH-DA staining (green) indicates an accumulation of ROS. The results revealed that Ses prevented ROS accumulation stimulated by PM_2.5_ in H9C2 cells, and a dose-dependent therapeutic effect was notably observed (Figures 6E, F). In addition, the damage to mitochondrial membrane potential caused by PM_2.5_ was remarkably reversed by Ses (Figures 6G, H). Collectively, these results indicate that Ses exerts strong antioxidant effects and inhibits mitochondrial damage in cardiomyocyte exposed to PM_2.5_.

### Ses attenuates ferroptosis caused by PM_2.5_ in H9C2 cells

**FIGURE 7:**
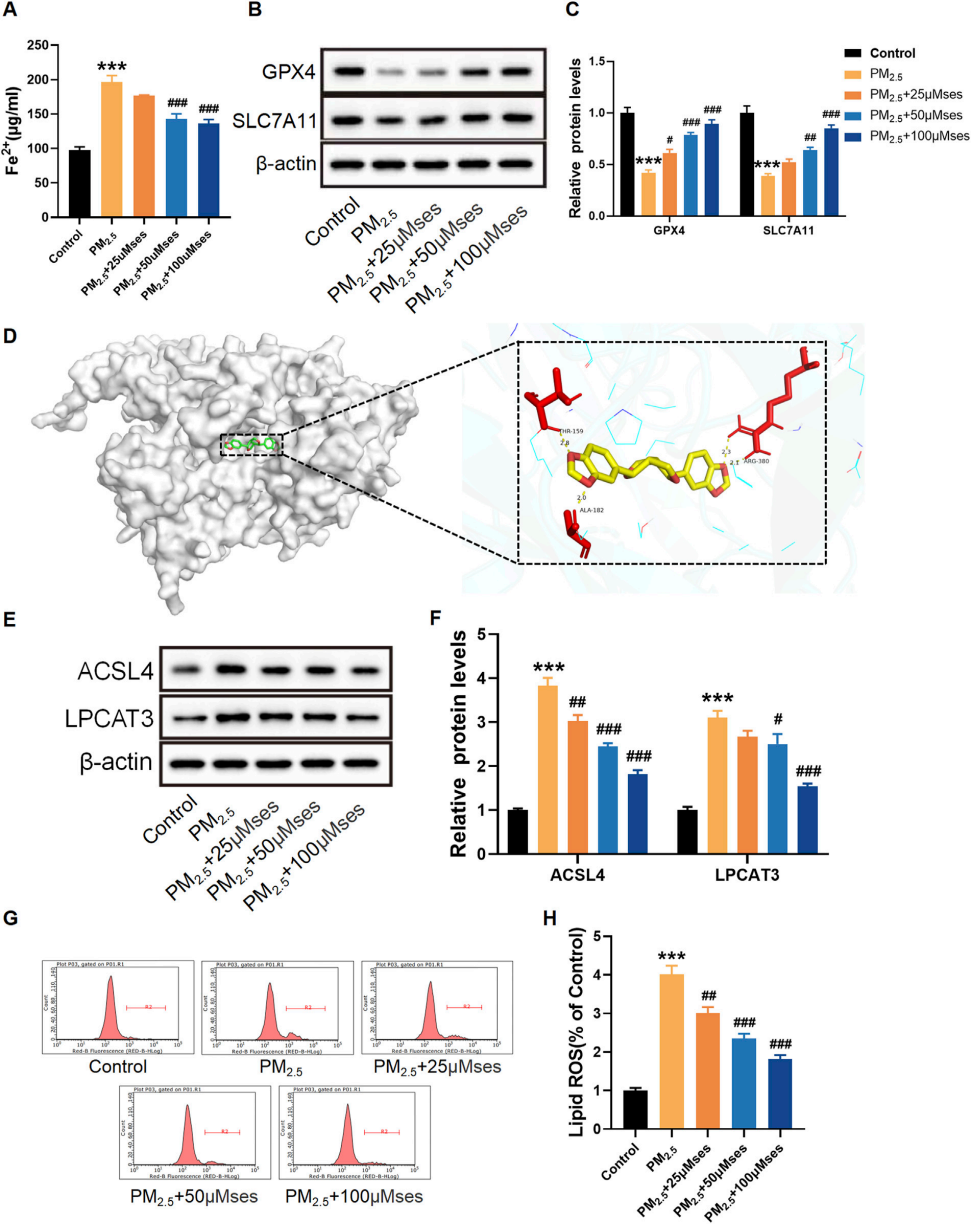
Sesamin (Ses) attenuates ferroptosis caused by PM_2.5_ in H9C2 cells. **(A)** Quantitative analyses of Fe^2+^. **(B)** GPX4 and SLC7A11 levels were examined by Western blot. **(C)** Quantitative analyses of GPX4 and SLC7A11. **(D)** Binding modes of Ses to ACSL4. **(E)** ACSL4 and LPCAT3 levels were examined by Western blot. **(F)** Quantitative analyses of ACSL4 and LPCAT3. **(G)** Lipid ROS with C11-BODIPY^581/591^ was measured by Flow cytometry. **(H)** Quantitative analyses of lipid ROS. The data were expressed as mean ± SEM. All datas were obtained from three replicate experiments. ***p < 0.001, *versus* the Control. *p < 0.05, *versus* the PM_2.5_ group. ^##^ *p* < 0.01, *versus* the PM2.5 group. ^###^ *p* < 0.001, *versus* the PM_2.5_ group.

In ferroptosis, iron accumulation and lipid peroxidation are the two most important signs. Consequently, reducing the excess iron content is crucial for restraining ferroptosis. The present results indicate that PM_2.5_ facilitates iron accumulation, whilst relatively high doses of Ses inhibit this progress (Figure 7A). The expression levels of ferroptosis-related proteins, including GPX4, SLC7A11, ACSL4 and LPCAT3, were measured to determine whether Ses inhibits cardiomyocyte ferroptosis. The results reveal that PM_2.5_ downregulated the expression of GPX4 and SLC7A11 in H9C2 cells, whereas Ses restored the expression of both proteins (Figures 7B, C). Through molecular docking, the interaction between Ses and ACSL4 was confirmed (Figure 7D). The binding sites between Ses and ACSL4 were THR-159, ALA-182 and ARG-380. The binding energy was −9.1 kcal/mol (<−5 kcal/mol), indicating a strong binding affinity between the ligand and the protein. Similarly, Ses downregulated the elevated levels of ACSL4 and LPCAT3 caused by PM_2.5_ (Figures 7E, F). C11-BODIPY^581/591^ is a fluorescent probe developed to detect lipid peroxidation. As shown in Figures 7G, H, the fluorescence signal intensity of the oxidised BODIPY remarkably increased after PM_2.5_ exposure, whilst it was reduced by Ses pretreatment.

## Discussion

The study provides evidence that Ses pretreatment exerts positive effects on PM_2.5_-triggered cardiovascular injuries by inhibiting ferroptosis. A potential approach for the prevention of CVDs was identified. Clinical and epidemiological studies indicate that exposure to PM_2.5_ increases the rate of cardiovascular death (Fiordelisi et al., 2017; Rajagopalan et al., 2018; Wang et al., 2019). However, scholarly investigations on the underlying mechanisms of CVDs due to exposure to PM_2.5_ are rarely conducted. Several studies indicate a link between ferroptosis and CVDs. Ferroptosis plays a crucial role in doxorubicin- and ischemia/reperfusion-induced cardiomyopathy (Fang et al., 2019), myocardial infarction (Wang and Kang, 2021) and heart failure (Liu et al., 2018). The focus of this study was the role of ferroptosis in PM_2.5_-triggered cardiovascular injuries. The results indicated that PM_2.5_ exposure prominently increased the level of oxidative stress and caused disorders of iron metabolism. Ses pretreatment may mitigate these changes induced by PM_2.5_. Consequently, Ses is considered beneficial for ferroptosis caused by PM_2.5_.

Cardiovascular events caused by PM_2.5_ may be indirectly attributable to the induction of oxidative stress (Kaihara et al., 2021). ROS plays a crucial role in oxidative stress (Li et al., 2021). In H9C2 cell experiments, previous studies have indicated that PM_2.5_ (Cao et al., 2016) and erastin (Li et al., 2020) can increase levels of ROS. However, research on the toxicity similarity between erastin and PM_2.5_ is still notably limited. In the present study, the morphology of H9C2 cells shifted clearly from fusiform to oblate in the erastin and PM_2.5_ group. Fer-1 pretreatment improved cellular morphology, restoring a spindle-like shape and clear cytoplasm. The findings indicate that PM_2.5_ has a detrimental impact on the activity of H9C2 cells, revealing that the ferroptosis inhibitor Fer-1 exhibited a repressive effect during this process. These results reveal that PM_2.5_ may exert a negative, erastin-like effect. LDH is a marker of cellular damage. As a stable cytoplasmic enzyme existing in all cells, LDH is released into the cell culture supernatant when the plasma membrane is impaired (Kumar et al., 2018; Ma et al., 2021). In the current study, the level of LDH remarkably increased after PM_2.5_ stimulation, and Fer-1 was able to attenuate this injury. PM_2.5_ can elevate ROS levels through the multiple pathways, such as Fenton reaction or the activation of inflammatory cells. Excessive ROS generation modifies the expression of antioxidant enzymes, such as SOD, GSH and GSH-Px (Ragab et al., 2022). The levels of MDA reflect the degree of lipid peroxidation and cellular damage caused by free radicals (Yang et al., 2021). In this study, the results showed that the activities of SOD, GSH and GSH-Px notably reduced in the PM_2.5_ exposure group, and Fer-1 attenuated these changes. PM_2.5_ exposure resulted in a remarkable increase in MDA content. However, Fer-1 diminished this change induced by PM_2.5_. The above results indicate that the ferroptosis inhibitor Fer-1 can alleviate the damage caused by oxidative stress.

Ferroptosis was initially identified as a novel mechanism of cellular demise, characterised by unique attributes that differentiate it from other modes of cell death. This distinctive type of cell death is primarily induced by iron-dependent phospholipid peroxidation. Inhibition of GPX4 or System xc^−^ can potentially lead to the accumulation of lipid peroxides, thereby triggering the process of ferroptosis. System x_c_^−^ is a transmembrane protein complex comprising two key subunits (SLC7A11 and SLC3A2). Cystine is transported from the extracellular space to the intracellular environment via System x_c_^−^. Once cystine is taken up into the cell, it is reduced to cysteine and utilised for the synthesis of GSH (Liu et al., 2022). Reduced (GSH) is converted to oxidised (GSSG) by GPX4 in the glutathione redox cycle (Shao et al., 2022). Previous research has shown that PM_2.5_ entering cells can lead to elevated levels of Fe^2+^, which may trigger the Fenton reaction. This reaction generates significant amounts of ROS, resulting in glutathione depletion, lipid peroxidation, and ultimately, ferroptosis (Guo et al., 2023). In our study, we also found that PM_2.5_ exposure notably decreased the expression of SLC7A11 and GPX4, whilst Fer-1 pretreatment increased their expressions. The results of the study indicated that targeted inhibition of ferroptosis could be a viable approach for the prevention and treatment of CVDs.

Some phytochemicals, such as resveratrol, quercetin and anthocyanins, have been reported to reduce the damage of PM_2.5_ on human health (Guo et al., 2024). However, most phytochemicals tend to have lower absorption rates in the human body due to their inherent properties, making them difficult to supplement directly through dietary intake. Ses, a lignan compound found in sesame seeds and oil, has been shown to have multiple health benefits. Previous studies have indicated that the absorption rate of Ses can exceed 90% (Umeda-Sawada et al., 1999), further emphasising its potential in improving human health. Li et al. found that taking Ses (50 and 100 mg) for 4 weeks can reduce mean pulmonary arterial pressure and right ventricular systolic pressure in monocrotaline-triggered hypertensive rats (Li et al., 2015). Loke et al. (Loke et al., 2010) also indicated that Ses can decrease the formation of atherosclerotic lesions. Despite the numerous therapeutic features of Ses, the persistence of the beneficial effects on PM_2.5_-triggered cardiomyocyte injury remains unclear. This study initially demonstrated that Ses substantially attenuated cellular damage and oxidative stress triggered by erastin. Based on these results, the protective effect of different Ses concentrations on cardiovascular injury triggered by PM_2.5_ was investigated. The results showed that Ses pretreatment remarkably reduced the levels of MDA and increased the activities of SOD, GSH and GSH-Px. The fluorescence intensity of ROS in the H9C2 cells subjected to PM_2.5_ treatment exhibited a remarkably greater magnitude compared to the control group. Dose-dependent differences were also observed in cells pretreated with different concentrations of Ses. The aforementioned results indicate that Ses pretreatment attenuates cellular damage and oxidative stress induced by PM_2.5_. The effects of Ses on ferroptosis-related proteins GPX4 and SLC7A11 were further evaluated. The results indicated that the expressions of SLC7A11 and GPX4 markedly decreased in the PM_2.5_ group, whilst Ses pretreatment restored their expression. These results reveal that Ses may act as a ferroptosis inhibitor to alleviate the toxicity of PM_2.5_ in cardiomyocytes.

As a key lipid metabolism enzyme, the acyl-CoA synthetase long-chain family member 4 (ACSL4) plays a crucial role in ferroptosis (Ji et al., 2022). Lysophosphatidylcholine acyltransferase 3 (LPCAT3) is a crucial factor in producing lipoprotein (Xu et al., 2022). Polyunsaturated fatty acids (PUFAs) are the principal substrates of lipid peroxidation in the development of ferroptosis. ACSL4 and LPCAT3 accelerate ferroptosis through the integration of PUFAs into cellular phospholipids (Dixon et al., 2015; Yuan et al., 2016; Doll et al., 2017; Kagan et al., 2017). The network pharmacology results revealed that ACSL4 is the core target for Ses to exert protective effects, ranking first based on the clustering coefficient scores of genes. Therefore, ACSL4 may be a potential target for Ses to alleviate PM_2.5_ cardiotoxicity. Western blot results indicated that the group of H9C2 cells treated with PM_2.5_ exhibited elevated ACSL4 levels when compared to the control group. However, Ses treatment reversed these changes. Additionally, the findings are further validated through molecular docking analysis. Studies have shown that Protosappanin A can physically bind with ferroptosis-related protein ACSL4, inhibiting its phosphorylation and subsequent phospholipid peroxidation. While also preventing FTH1 autophagic degradation and subsequent release of ferrous ions (Fe^2+^) (Cui et al., 2024). This may also be a possible mechanism by which Ses exerts its anti-ferroptotic activity.

Overall, Ses was found to attenuate PM_2.5_-triggered cardiomyocyte injury. The potential mechanisms of Ses supplementation to mitigate PM_2.5_-induced cardiomyocyte damage through the targeting of ACSL4-mediated ferroptosis. Hence, pursuing nutritional interventions that target ACSL4-mediated ferroptosis presents a promising approach for developing effective countermeasures against PM_2.5_-induced cardiomyocyte injury.

## Data Availability

Publicly available datasets were analyzed in this study. This data can be found here: https://www.ncbi.nlm.nih.gov/geo/query/acc.cgi?acc&equals;GSE211949; (GSE211949).
