# Supplementary material for: Exploring the mechanism of sesamin for the treatment of PM2.5-induced cardiomyocyte damage based on transcriptomics, network pharmacology and experimental verification
Source: Front Pharmacol. 2024 Nov 5;15:1486563. doi: 10.3389/fphar.2024.1486563 (PMC11573564; doi:10.3389/fphar.2024.1486563)
Supplement: Supplementary file 1 [file Table1.DOCX]

**Table S1** DEGs involved in Ferroptosis

| **Uniprot** | **Gene** | **Description** |
| --- | --- | --- |
| Q9QUJ7 | Acsl4 | Long-chain-fatty-acid--CoA ligase 4 |
| Q61147 | Cp | Ceruloplasmin |
| P09528 | Fth1 | Ferritin heavy chain |
| P14901 | Hmox1 | Heme oxygenase 1 |
| Q91VR7 | Map1lc3a | Microtubule-associated proteins 1A/1B light chain 3A |
| P04925 | Prnp | Major prion protein |
| Q75N73 | Slc39a14 | Metal cation symporter ZIP14 |
| Q62351 | Tfrc | Transferrin receptor protein 1 |
| Q921I1 | Trf | Serotransferrin |
| Q60931 | Vdac3 | Voltage-dependent anion-selective channel protein 3 |
